# Completely engaged three-dimensional mandibular gear-like structures in the adult horned beetles: reconsideration of bark-carving behaviors (Coleoptera, Scarabaeidae, Dynastinae)

**DOI:** 10.3897/zookeys.813.29236

**Published:** 2019-01-07

**Authors:** Wataru Ichiishi, Shinpei Shimada, Takashi Motobayashi, Hiroaki Abe

**Affiliations:** 1 Department of Biological Production, Faculty of Agriculture, Tokyo University of Agriculture and Technology, Saiwai-cho 3-5-3, Fuchu, Tokyo 183-8509, Japan Tokyo University of Agriculture and Technology Tokyo Japan

**Keywords:** beetle, bark-carving behavior, gear-like structure, horned beetle, mandible

## Abstract

Adult horned beetles (Coleoptera, Scarabaeidae, Dynastinae) such as *Trypoxylusdichotomus* (Linnaeus, 1771) exhibit bark-carving behaviors to feed on tree sap, in part by using small projections of the clypeus. However, in the present experiments, adult horned beetles (*T.dichotomus* and *Dynasteshercules* (Linnaeus, 1758)) used their mandibles and not the projections of the clypeus to carve bark. Our findings show the presence of completely engaged mandibular interlocking, gear-like surface structures in molar areas that guide mandible opening and closure, and lead to completely synchronous movements of adult horned beetle mandibles. Three-dimensional shapes of these mandibular gear-like structures are complex and remained in contact after the death of a beetle. Moreover, adult horned beetles often performed bark-carving behaviors using only the mandible of one side, suggesting that the primary role of the mandibular gear-like structure is to prevent breakage of the mandible by transmitting load from one mandible to the other. Among the 22 Dynastinae and 16 other beetle species examined (not Dynastinae), the gear-like structure was found in all the Dynastinae species and in no other species.

## Introduction

Horned beetles (Coleoptera, Scarabaeidae, Dynastinae), including those of the genera *Dynastes* (Linnaeus, 1758), *Megasoma* (Linnaeus, 1758), *Chalcosoma* (Linnaeus, 1758), *Eupatorus* (Hope, 1831), and *Trypoxylus* (Linnaeus, 1771), are mostly large insects. Adults are typically sexually dimorphic and males have long horns extending from the head and thorax, whereas females have no horns. The largest *Dynasteshercules* (Linnaeus, 1758) male adults measure more than 160 mm from the tip of the thoracic horn to the end of the abdomen (Imamori 2011) (Suppl. material 1). Adults feed on the sap of wounded trees, whereas horned beetle larvae feed on rotten wood and wood litter. Horned beetles are imported to Japan from foreign countries as popular insect pets, and numerous books describe corresponding characteristics and breeding methods (Unno 2006, McMonigle 2012). Therefore, the life cycle, behavior, and breeding methods of horned beetles are well known in Japan. However, little is known about the mouthparts of horned beetles.

**Figure 1.**
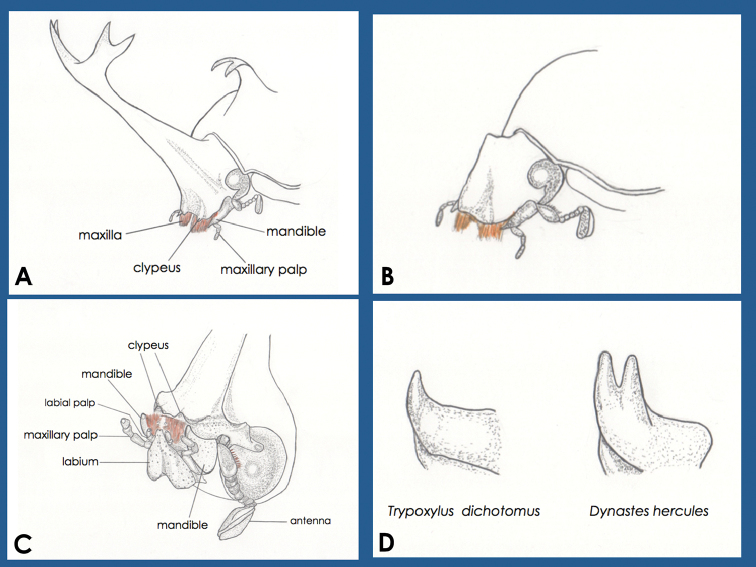
Diagrams of the mouthparts of adult horned beetles. **A** from an oblique anterior side of a *Trypoxylusdichotomus* male **B** from an oblique anterior side of a *T.dichotomus* female; names of mouthparts were omitted **C** View from an oblique ventral side of *T.dichotomus* male **D** diagram of two types of adult mandible tips; left, the tip of the left mandible of a *T.dichotomus* male from the left side; this tip does not branch off; right, the tip of a left mandible of a *Dynasteshercules* male from the left side; this tip branches off and is forked.

The horned beetle species *Trypoxylusdichotomus* (Linnaeus, 1771) is very common in Japan (Suppl. material 1), and numerous individuals are present in Satoyama, which is an area between mountain foothills and arable flat land. In Satoyama, many *Quercusacutissima* and *Q.serrata* are planted for use as bed logs in traditional mushroom cultivation. *Trypoxylusdichotomus* larvae feed on the rotten wood and wood litter from these trees. Adults of this species emerge in the summer and feed on the sap from tree wounds that are thought to be made by boring insects such as the larvae of cossid moths (Yoshimoto and Nishida 2007). Adult *T.dichotomus* feed extensively on sap and bark-carving behaviors have been observed on *Fraxinusgriffithii* trees (Hongo 2006), and these involved repeated head-scooping movements. Because the surfaces of *F.griffithii* trees are soft, adult *T.dichotomus* likely carve soft tree bark to obtain sap. In addition, previous real-time macroscopic video observations suggested that adult *T.dichtomus* carves bark using the projection of the clypeus, which is a chisel-like feature on the front of the head (Hongo 2006) (Fig. 1).

Under rearing conditions, when the small plastic food cup becomes empty, beetles exhibit bark-carving behavior to obtain more sap (in this case, jelly). These activities have been observed in nature, as telecast by Nippon Hoso Kyokai (NHK) in their 5 August 2007 telecast “Darwin has come! The king of beetles! Hercules”. In their televised animation, adult *D.hercules* reportedly start by biting the bark with the mandibles to soften the tree and then use the spatula-like projection of the clypeus to carve the softened bark.

Mouthpart forms vary greatly between insect species and are related to the diet but are classified into two basic types: one adapted for biting and chewing solid food (mandibulate) and the other adapted for sucking fluids (suctorial or haustellate) (Chapman 2013). Beetle mouthparts are much more uniform and are mostly of the biting-chewing types. Mandibles, or jaws, are shaped according to the food consumed by the insect (Nel and De Villiers 1988, Nel and Scholtz 1990, Sanderson and Jackson 2001). Many adults of lepidopteran species develop a proboscis and have mandibles that are either nonfunctional, vestigial, or absent (Grimaldi and Engel 2005). In some enlarged jaw forms, such as those of adult male stag beetles (Lucanidae), huge mandibles do not contribute to feeding (Snodgrass 1993, Goyens et al. 2014, Goyens et al. 2016). The biting type, as found in grasshoppers and caterpillars, is the more typical, and the mandibles comprise single tooth-like pieces and move in a transverse plane (Sanderson and Jackson 2001, Clissold 2007). Moreover, as in mammalian anatomy, the more distal part of the mandible has a cutting function and is referred to as the incisor region, whereas the proximal part often has a grinding function and is referred to as the molar region (Chapman 1995). In recent studies of the mouthparts of insects, X-ray micro-CT analysis was used to investigate the internal morphology of insect heads without dissection or damage (Li et al. 2011, Beutel et al. 2014, Weihmann et al. 2015). In contrast, because the mandibles of horned beetles are small and inconspicuous, they have never been studied in detail.

Mouthparts of adult *T.dichotomus* horned beetles are illustrated in Fig. 1 and shows the presence of small and obscure mandibles on both sides of the head. The maxillae of adult horned beetles have orange brush shapes. In adult horned beetles, these maxillae are more outstanding than the mandibles (McMonigle 2012). Our observations indicate that projections of the clypeus in adult *T.dichotomus* or *D.hercules* are not sharp, and that intense bark carving may injure mouthparts such as the labrum.

Herein, we used video footage to study the relationship between bark-carving behaviors and structures of mouthparts of horned beetles, particularly those of *T.dichotomus* and *D.hercules*. In field experiments, angles and directions of video footage are restricted from dorsal or lateral sides because adult *T.dichotomus* and *D.hercules* hold on to the tree. Moreover, these beetles bury their head into the wounds of trees, precluding detailed filming of the mouthparts during bark-carving. Because *T.dichotomus* behaviors were analyzed in a seminal study (Hongo 2006) and *D.hercules* behaviors were telecast by NHK, we filmed bark-carving behaviors under controlled laboratory conditions using adult *T.dichotomus* and *D.hercules*, which are easy to breed in sufficient numbers for experiments.

Our observations indicated that *T.dichotomus* and *D.hercules* use their mandibles like a chisel to carve bark and do not use the projection of the clypeus (Suppl. materials 1–3). Moreover, *T.dichotomus* and *D.hercules* did not bite the bark by opening and closing of their mandibles. Furthermore, we observed completely synchronous movements of both mandibles. In subsequent analyses of the insect head, mandible movements were simultaneous and their asymmetrical hind regions resembled a gear structure. Approximately 1,700 species of horned beetles have been identified globally (Kohiyama 2014) and, although horn shapes and lengths in male adults vary between species, mouthpart structures are very similar. Thus, to determine whether gear-like structures of mandibles are common to these adult horned beetles, we examined 22 Dynastinae and 16 other beetle species (not Dynastinae). Our studies show that mandible gear-like structures are present only in the examined adult Dynastinae and are not present in 16 other beetle species.

## Materials and methods

### Insects

Among twenty-two horned beetle species three were maintained at the Tokyo University of Agriculture and Technology: *Trypoxylusdichotomus* (adult male length, 40–80 mm; adult female length, 40–60 mm (Bouchard et al. 2014)), *Dynasteshercules* (♂ 50–170 mm; ♀ 40–60 mm (Bouchard et al. 2014)), and *Endebiusgideon* (Linnaeus, 1767) (body length, 35–75 mm (Bouchard et al. 2014)). The other horned beetle species *Chalcosomaatlas* (Linnaeus, 1758) (60–130 mm (Bouchard et al. 2014)), were purchased from Fujikon Co., Ltd. (http://www.fujikon.net/), Lumberjack Co., Ltd. (http://www.lumber-j.com/) and various insect-dealing internet sites (for example http://64auc.sakura.ne.jp/):*Chalcosomachiron* (Oliver, 1789) (50–130 mm (Kohiyama 2014)), *Megasomaelephas* (Fabricius, 1775) (58–130 mm (Kohiyama 2014)), *Megasomaactaeon* (Linnaeus, 1758) (50–135 mm (Kohiyama 2014)), *Dynastestityus* (Linnaeus, 1763) (40–60 mm (Bouchard et al. 2014)), *Dynastesneptunus* (Quensel, 1817) (50–150 mm (Kohiyama 2014)), *Dynastessatanas* (Moser, 1909) (55–115 mm (Kohiyama 2014)), *Eupatorusbirmanicus* (Arrow, 1908) (40–50 mm (Kohiyama 2014)), *Haploscapanes Barbarossa* (Fabricius, 1775) (26–56 mm (Kohiyama 2014)), *Allomyrinapfeifferi* (Redtenbacher, 1867) (27–40 mm (Kohiyama 2014)), *Augosomacentaurus* (Fabricius, 1775) (33–80 mm (Kohiyama 2014)), *Oryctesrhinoceros* (Linnaeus, 1758) (30–45 mm (Unno 2006)), *Eophileuruschinensis* (Faldermann, 1835) (18–25 mm (Kohiyama 2014)), *Golofaaegeon* (Hope, 1837) (58 mm (Imamori 2011)), *Golofapizarro* (Hope, 1837) (25–40 mm (Unno 2006)), *Endebiusflorensis* (Lansberge, 1879) (77 mm (Imamori 2011)), *Eupatorusgracilicornis* (50–90 mm (Kohiyama 2014)), *Cyclocephalacomplanata* (Burmeister, 1847) (16 mm), and *Hexodonlatissimus* (Olivier, 1789) (28 mm). The body weight of an adult male *D.hercules* was 22 g and adult female weighed 18 g, whereas the *T.dichotomus* adult male weighed only 8 g (Suppl. material 1).

*Dynasteshercules* are naturally found in the Neotropical region of southern Mexico to Bolivia and Trinidad, Guadeloupe, and Dominica in the West Indies (Ratcliffe and Cave 2015). *Dynasteshercules* are among the largest beetles and are the most recognizable insect species in the world. Adults fly mostly at night, especially during the first two hours after sunset (Bouchard et al. 2014). *Trypoxylusdichotomus* are the largest and best-known beetles in Japan. Adult males of this species use their long, forked horns in battles with rival males. Males are active all summer long, whereas females die soon after laying their eggs (Bouchard et al. 2014).

All larvae of horned beetles were fed on breeding fermentation mats (DEBURO Pro) that were purchased from Fujikon Co., Ltd. (http://www.fujikon.net/). All adults were fed on breeding jelly (DORCUS JELLY) containing animal protein, trehalose, collagen, and banana flavor (Fujikon Co., Ltd).

Synchronous movements and gear-like structures were characterized and compared between adult beetles and adults of various other insect species. Experiments were performed with five males and five females of *T.dichotomus* and *D.hercules*, and comparisons were made with single adult males of all other horned beetle species. Sixteen adult specimens of other beetle species (not Dynastinae) were collected from the field or purchased from insect-dealers. Sexes of these non-horned beetles were not recorded. Mouthparts of insect specimens were softened by immersion in water all day prior to observations of the synchronous movements and the gear-like structures of mandibles.

### Observations of the mandible muscles

To observe the adductor and abductor muscles of the mandible, adult *T.dichotomus* specimens were fixed and preserved in 90% alcohol. Heads were dissected carefully using a file (Bkong YWE-B pencil type router; Yanase Corp., Hyogo, Japan) and tweezers. Mandible muscles were observed using binocular microscopy (Stemi 2000-C; Carl Zeiss Microscopy Co., Ltd.). We compared apparent volume of photographed adductor and abductor muscles. Ratios of adductor and abductor muscles in *T.dichotomus* biting type mandibles were compared with those of adult *Locustamigratoria* (Linnaeus, 1758) (Orthoptera: Acrididae).

### Measurements of mandible strengths and resistance to breakage

**Figure 2.**
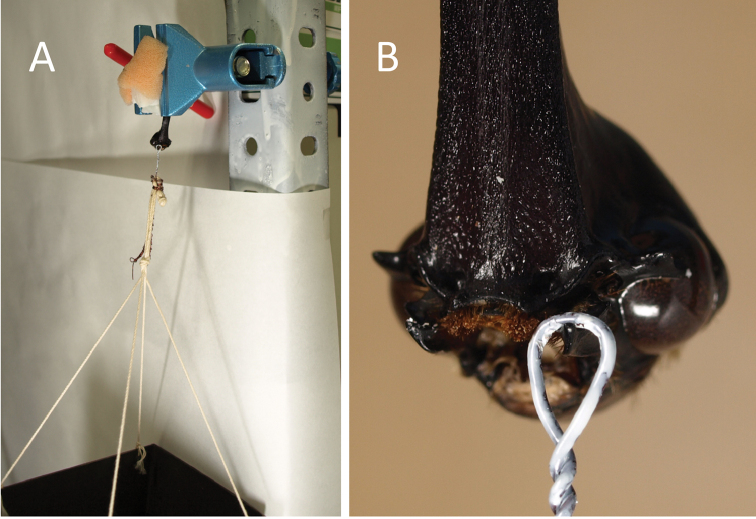
Measurements of mandible strengths of break resistance. **A***T.dichotomus* horns were fixed in a small vice **B** wire was hung on the tip of the mandible.

Initially, horned heads were separated from dead male *T.dichotomus* specimens and were soaked in tap water overnight to soften the hardened articulations, and the labium and maxillae were then removed carefully using a file and tweezers. The average length from the tip of the horn to bottom of the head was 29.3 mm (Suppl. material 4). Twenty male heads with intact gear-like structures between right and left mandibles were used for measurements of right and left mandible strengths in break resistance analyses. Similarly, ten male heads with broken gear-like structures were used in tests of strengths according to breakage resistance. Head specimens were then fixed by sandwiching the horns between two plastic erasers (to prevent slipping) using a sponge and a small vice as shown in Fig. 2A. A paper container with a wire was attached to grip the tip of the mandible (Fig. 2B) and metal nuts of 4.5 g were used as weights. Load was applied to the mandibles at a right angle to the rotary direction of movement (Suppl. material 5). The container wire was then hung on the tip of a single mandible and the metal nuts were added stepwise to the container until the mandible was dislocated and fell from the mouth. Gross weights of nuts and the container were then recorded. Statistical analyses were performed using a generalized linear model (GLM) with R (Version 3.2.2). For multiple comparisons, Tukey-Kramer tests were used to identify differences between groups of mandibles.

### Paraffin surface scratching tests with horned beetle heads

**Figure 3.**
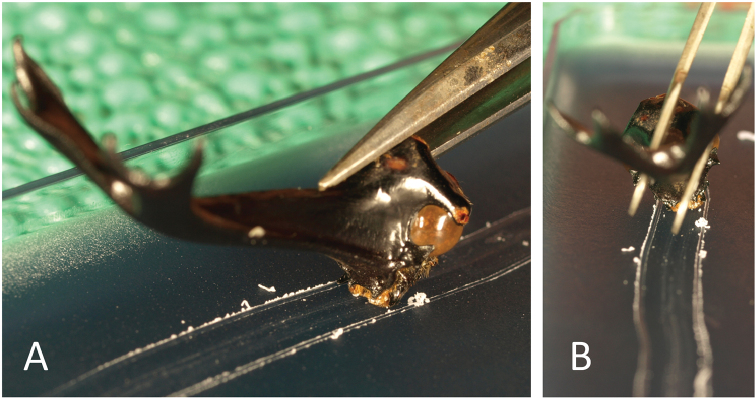
Scarring of flat paraffin surfaces; *T.dichotomus* heads were placed in the back position to show the widths of scars. **A** lateral view **B** frontal view. Only scratches from *T.dichotomus* are shown.

If horned beetles use mandibles to carve bark, the tips of the mandibles must be in contact with the tree surface earlier than the projections of the clypeus when the beetle takes the posture for bark carving. Therefore, we determined whether the tips of the mandibles or the projections of the clypeus touch the tree surface first. To this end, paraffin (solidification point about 51–53 °C) was melted, and a small piece of black crayon was melted and mixed in the plastic container to color the paraffin (Sterile No. 2 Square Schale; Eiken Kizai Co., Ltd. Tokyo, Japan), and was then cooled until it hardened. Initially, we determined whether tips of mandibles or the projections of clypeus made pits in the paraffin surface first. But pits on the paraffin were harder to see than scratches on the paraffin. Thus, heads of horned beetles of several genera were manually held at various forward angles (from 0°to 60°) between the horn and paraffin and were used to scratch the paraffin surface. Heads were also placed in the back position and the scars were photographed (Fig. 3).

### Video recording and mandible gear-like structures filing procedures

Videos of bark-carving behaviors and actions of mandibles were recorded using a Sony Handycam HDR-PJ590V digital HD video recorder (Sony Corp., Tokyo, Japan). Perches comprised rotten wood and plates of jelly, which were made from logs that were used for cultivation of mushrooms, and were purchased from Fujikon Co., Ltd. We assumed that the bark-carving behavior observed in laboratory conditions on wood logs is similar to that observed on tree barks in the wild. Videos of bark-carving behaviors were recorded using adult *D.hercules* and *T.dichotomus*. The carving behavior of an adult male *T.dichotomus* was also recorded in a plastic green insect cage. To obtain specimens with broken gear-like structures between mandibles but no damage to articulation, gear regions of mandibles were disabled using a file (Bkong YWE-B pencil type router; Yanase Corp., Hyogo, Japan). The router bit was made from a nail and acrylic resin was used to bind mandibles. All materials were purchased from Fujikon Co., Ltd. (http://www.fujikon.net/) as an insect specimen kit.

## Results

### Bark-carving behaviors and mandibles

**Figure 4.**
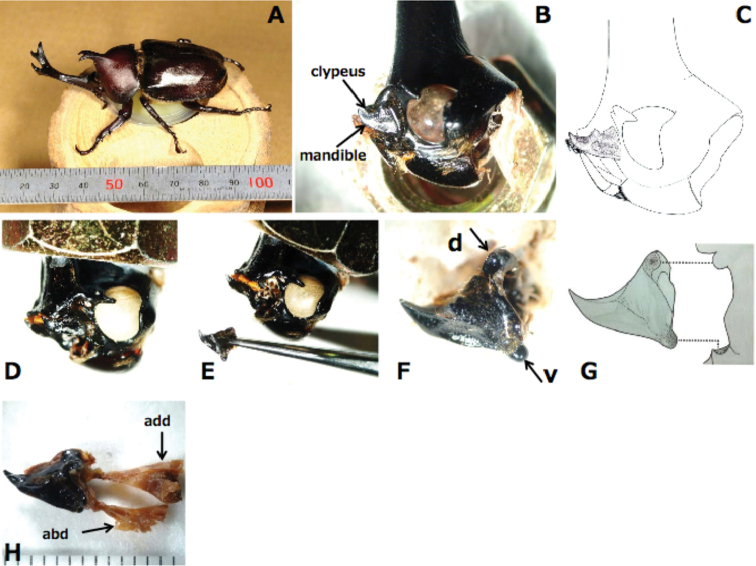
Mandibles of *T.dichotomus* adults. **A** A male adult viewed from the left side with a ruler **B** lateral view of the head from the left side; the clypeus and mandible are indicated by arrows. Antenna and maxillary palp parts were removed to expose the mandible **C** diagram of the head; the mandible is indicated by a stipple **D** a head before removing the mandible **E** a head after removing the mandible **F** the left side mandible was dissected from the cranium **G** diagram showing the double articulation of the mandible; the right side diagram is of the cranium **H** a left side mandible of *T.dichotomus* with attached muscles. Abbreviations: add = adductor muscle; abd = abductor muscle; d = dorsal articulation (socket); v = ventral articulation (condyle). Scale bar: 1 mm.

**Figure 5.**
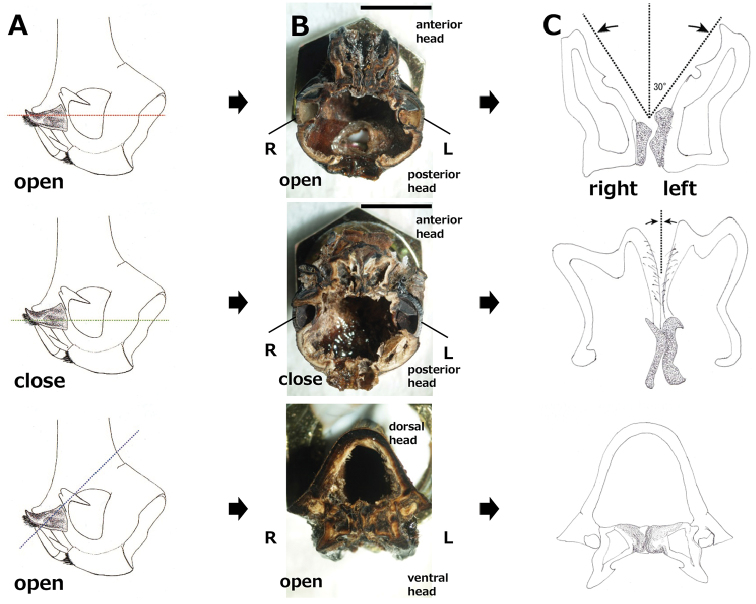
Engagement of gear-like structures on mandibles of *D.hercules* during opening and closing; engaged regions of mandibles were exposed using a file. **A** Lateral view of the head from the left side; dotted lines indicate the position of the transverse section **B** interior view of the head from the ventral side **C** diagram of the engagement region; gear-like structures are emphasized by stipple. Abbreviations: R = right compound eye; L = left compound eye. Scale bar: 7 mm.

Mandibles of adult horned beetles are present on both sides of the head (Figs 1, 4A–C). Two types of mandible tips have been described previously (Rowland and Miller 2012); those in *Chalcosoma*, *Eupatorus*, and *Trypoxylus* do not branch off, are sharp, and turn upwards, and those of *Dynastes* and *Megasoma* species are shaped like forks and turn upwards (Fig. 1 and Suppl. пaterial 6). No sexual differences in mandible forms were identified. Because mandible tips fail to make contact with each other, the mandibles of *T.dichotomus* and *D.hercules* do not have biting and chewing functions. The videos recorded in this study indicate that *T.dichotomus* and *D.hercules* use their mandibles to carve bark and do not use the projection of the clypeus. Use of the projection of the clypeus to carve bark or xylem would obscure the orange hair of the maxilla and labrum under the wood. However, in the images of adult male (from 20–40 s in Suppl. material 2 and 50 s to 1 min and at 30 s in Suppl. material 3), female *D.hercules* carving wood (from 1 s to 1 min and 10 s in Suppl. material 7) and adult male *T.dichotomus* beetles carving the green plastic cage (from 1 min and 28 s to 1 min and 35 s in Suppl. material 7), the orange hairs are visible. Moreover, during bark-carving, mandibles were fully opened (Suppl. materials 2, 3, 7) to about 30 degrees from the closed position (Fig. 5A–C), and microscope observations show that the tips of the mandibles were projected further forward than the projection of the clypeus. To confirm these observations, we scratched a flat paraffin surface using the heads of various horned beetle genera at various forward-position angles. In all cases, mandibles, rather than projections of the clypeus, scratched the paraffin. We showed only the scars from paraffin surface scratching experiments with *T.dichotomus* in Fig. 3.

### Gear-like structures on mandibles

In further experiments, both mandibles of adult horned beetles moved in complete synchrony. Specifically, manual opening and closing of the right mandible of living adult *T.dichotomus* and *D.hercules* beetles were accompanied by synchronous opening and closing of the left mandible. Similarly, movements to the left and right were precisely synchronous in both mandibles, and simultaneous mandible movements were also observed in dead insects (Suppl. material 8). These observations suggest that the mandibles are mechanically connected.

To investigate mechanical connections between left and right mandibles, we dissected the heads of *T.dichotomus* and *D.hercules* and observed mandible structures under a stereomicroscope. Mandibles of adult *T.dichotomus* and *D.hercules* are single, heavily sclerotized pieces with a dicondylic articulation. The ventral condyle of the mandible is a ball-like structure that fits into a socket like the acetabulum of the cranium, and the dorsal condyle of the cranium fits into an acetabulum of the mandible (Fig. 4D–G). This dicondylic articulation is typical for biting-chewing mouthparts. Because two points of the articulation are central to the axis of the mandibular movement, *T.dichotomus* and *D.hercules* mandibles can only move in a single plane. No sexual differences in mandible forms were identified. Mandibular muscles differed from those of typical biting-type insects. In particular, the adductor muscle of biting insects is generally very large and the abductor muscle is small (Chapman 2013, Snodgrass 1993), whereas in *T.dichotomus*, the adductor muscle is relatively small and the abductor muscle is larger than in other biting-type insects (Fig. 4H). Adductor and abductor muscles of mandibles are compared between *T.dichoromus* and typical biting type insect *L.migratoria* in Suppl. material 9. Furthermore, the posterior regions of mandibles that could not be observed from the outside were asymmetrical, and the gear-like structure was hard and engaged between the mandibles. Artificial devices that transmit force include V-belts and gears. The general definition of a gear is “a toothed machine part that engages successively with another toothed part to transmit motion or to change speed or direction.” Although the mandibles of *T.dichotomus* and *D.hercules* do not rotate 360°, their structure fulfills this definition, as observed in the mouth cavity after stripping the labium and maxillae. Initially, we suspected that these posterior region structures were molar cusps. However, adults of *T.dichotomus* and *D.hercules* could not separate engaged mandible and these gear-like structures remained in contact after death (Suppl. material 8). In contrast, no gear structures of mandibles were observed in larvae of *T.dichotomus* (Suppl. material 10) and *D.hercules* (Suppl. material 11), and posterior regions of mandibles in these specimens were separable, indicating that the gear-like structures appear at the adult stage.

**Figure 6.**
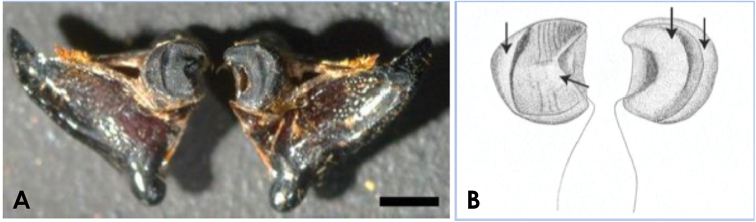
Gear-like structures on mandibles of adult horned beetles. To show the mandibular gear-like structure, the head was split along the anterior midline, and the mandibles were laid out and viewed from the inside. **A** Mandibles of a *T.dichotomus* male adult **B** diagram of structures in Fig. 6A; gear-like structures are shown as stipple. Arrows indicate the tip of each tooth. Scale bar: 2 mm.

The three-dimensional shape of the mandible gear-like structure is very complicated (Fig. 6A, B). Because the gear-like structure with the engagement of mandibles was difficult to represent in two dimensions, we solidified the mandibles of *D.hercules* by filling with acrylic resin and then filed the solidified mandibles to expose horizontal and ventral sections. As shown in Fig. 6, left and right mandibles have two-gear teeth each, and the engagement point moved with opening and closing of mandibles (Suppl. material 8).

In further analyses, we observed gear-like structures of mandibles in adults of 20 other species of horned beetle, suggesting that the gear-like structure of mandibles is common to all adult horned beetles (Suppl. material 6).

Many insects have asymmetric mandibles during the larval or adult stages (Snodgrass 1993). Thus, we examined the posterior regions of the mandibles from various other insect species, but found neither synchronous movements nor mandible gear-like structures (Suppl. material 6).

### Measurements of mandible break-resistance strengths

**Figure 7.**
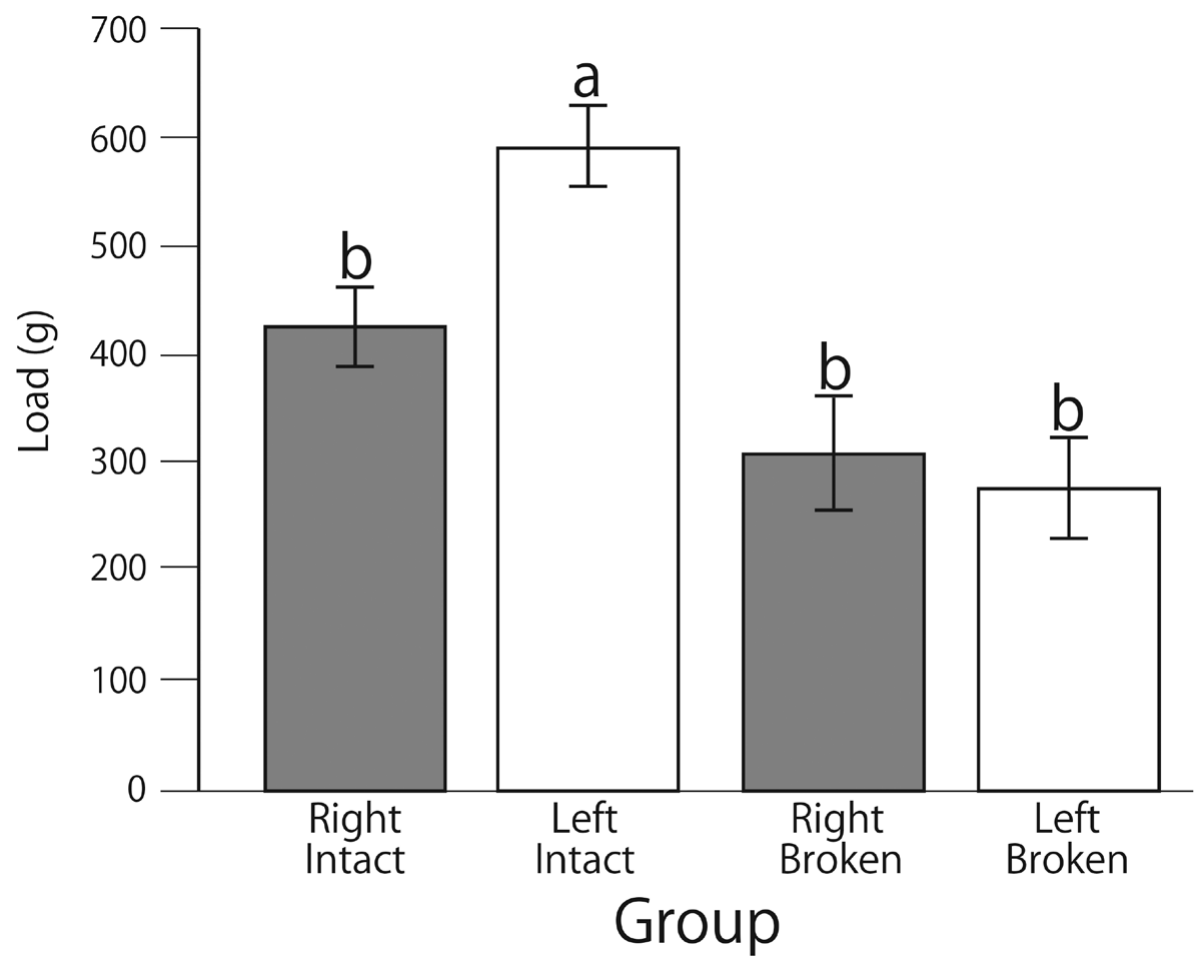
Load strengths of mandibles from a *T.dichotomus* adult male. Abbreviations: Right = right mandible; Left = left mandible. Intact = right and left mandibles in the intact state; Broken = the gear-like structure was broken before the measurement. Vertical bars indicate standard errors of the mean (SE). Significant differences between mandible break-resistance strengths are indicated by different letters (a, b; Tukey, p < 0.05).

To investigate the roles of the mandible gear-like structures, we measured the break-resistance strengths of mandibles from dead adult *T.dichotomus* under load. Preliminarily, we noticed that considerable force was necessary to dislocate single sides of intact and engaged mandibles from the cranium using tweezers. Moreover, although two articulations remained in the mandibles, these could be dislocated easily. However, there was a possibility that initial loading may damage the remaining mandibles and its articulations. Therefore to exclude this possibility, we used one intact specimen for only one side mandible dislocation experiments (Fig. 7, Right, Intact and Left, Intact groups). Additionally, to obtain the specimen in which the gear-like connection between mandibles was broken without damage to articulation, we filed the gear-like structure region (Suppl. material 12) and performed further load-dislocation experiments. As shown in Fig. 7 (Left, Intact), a load of 600 g was required to dislocate the left side mandible from the cranium. On the other hand, a load of about 400 g was required to dislocate the right side mandible. Thus, we found that the break-resistance strengths of right and left mandibles differed in the intact state (Fig. 7). Disengaged mandibles were dislocated under smaller loads than those of required to dislocate engaged mandibles (Fig. 7, Suppl. material 4). Although no significant differences were observed between “Right”, “Intact”, and “Broken” mandibles, disengaged right mandibles (Broken) were dislocated under smaller load than those required to dislocate engaged mandibles (Intact). In contrast, there were significant differences between “Left”, “Intact”, and “Broken” mandibles. Moreover, the difference in strength between right and left mandibles was not observed after filing the gear region (Fig. 7, Right, Broken and Left, Broken groups). Finally, unlike man-made single gear structures that do not prevent movement up and down along the axis of rotation, the complicated gear-like structure of horned beetle mandibles prevents such movements during the use of only one mandible side (Fig. 5C).

## Discussion

Adult *T.dichotomus* beetles exhibit bark-carving behaviors, and it is widely believed that small projections of the clypeus are involved in the process (Hongo 2006, Hongo 2012, Yagihashi et al. 2014). Our experiments and observations show that adults *T.dichotomus* beetles use their mandibles rather than the projection of the clypeus during bark-carving. We also demonstrate that adults *D.hercules* beetles use their mandibles to carve bark (Suppl. materials 2, 3, 7) and have completely engaged mandibular gear-like structures (Fig. 3, Suppl. material 8). These gears operate in two directions (open and close) and produce completely synchronous movements (Suppl. material 8).

Because mandible articulations of adult *T.dichotomus* and *D.hercules* beetles are placed under a considerable load during bark carving, this gear-like structure may primarily prevent the breakage of the mandibles. Accordingly, adult *D.hercules* exhibited bark-carving behaviors using only the mandible of one side, suggesting that the gear-like structure transmits the load from one mandible to the other, thus reducing the load on the mandible in use.

**Figure 8.**
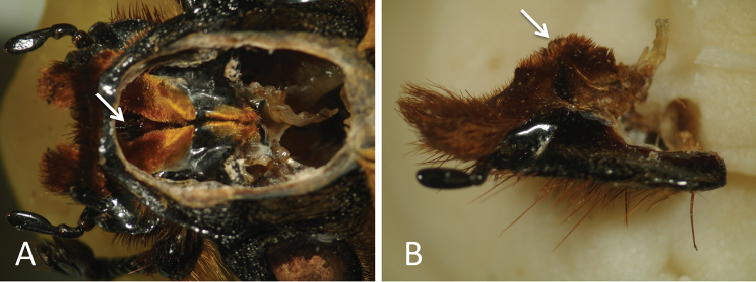
Mandibles and labium of *D.hercules*; engaged regions of mandibles were exposed using a file. **A** Interior view of the male head from the dorsal side; arrows indicate projections from the labium into the mouth cavity **B** lateral view of labium from the left side; arrows indicate projections into the mouth cavity.

Although synchronous movements of both mandibles may enhance break resistance strength, this is likely an insufficient explanation for the evolutionary conservation of moving functions in adult horned beetles. In addition to carving, mandibles of *T.dichotomus* and *D.hercules* are likely to facilitate sucking of sap as indicated by the narrow mouth cavity. After closing both mandibles, the inside regions with orange hair form a thick sandwich with projections from the labium into the mouth cavity (Suppl. material 13, Fig. 8). Moths and butterflies possess a proboscis (sucking tube) adapted to sucking fluid (nectar and sap). In the case of *T.dichotomus* and *D.hercules*, which do not have tubes, the beetles close their mandibles to create a narrow straw-like opening through which they suck the sap, which flows easily up the narrow mouth cavity and then down the alimentary canal. In accordance with the present observations of mandibular engagement, this function does not require skillful or independent movements of horned beetle mandibles. Moreover, following injury of muscle or nervous connections of one mandible, synchronous movements of both mandibles would be preserved by the gear-like structure. In addition, it was difficult to remove the labium due to its hardness and highly sclerotized state. We suggest that it protects the mouthparts, especially the maxillae.

Among very few studies of mandible movements in insects, investigations of the desert locust *Schistocercagregaria* (Orthoptera: Acrididae) showed that the mandibles of the two sides predominantly move together due to the synchronized activities of the adductor muscles shown in analyses of the sensory inputs required to maintain and change mandibular activities (Chapman 1995, Seath 1977a, Seath 1977b, Rast and Brauning 2001). In addition, *Odontomachus* ants (Hymenoptera: Formicidae) reportedly move both mandibles synchronously, and the required motor neuron circuits also allow independent mandible movements under certain conditions. *Odontomachus* ants move and control mandibular forces and speeds skillfully to perform various functions, including food consumption, brooding, and grooming (Just and Gronenberg 1999). It is thought that all ants use their mandibles skillfully (Holldobler and Wilson 1990). Hence, ant mandibles have evolved independence of left and right to enable skillful movements. In contrast, adult horned beetles do not move their mandibles skillfully and have small heads relative to their bodies. Horned beetle mandibles also have small adductor muscle quantities, suggesting that biting functions have been abandoned in favor of space in the head cavity. This may be advantageous for horned beetles, as they can bury their small heads into small wound to exude sap. Hence, mandibles of horned beetles may have evolved to have strength for dislocating load rather than skills by adopting a gear-like structure.

Currently, approximately 1,700 species of horned beetles have been identified in forests globally and most of these are in Southeast Asia and South America (Kohiyama 2014). Although horned beetle species vary in shapes and lengths of their adult horns, the mouthparts bear close resemblance to each other. Even between different species, the heads of the adult female horned beetles closely resemble each other. Moreover, the fundamental structural features of mandible gear-like structures were common in the present 22 species. Horned beetles have evolved into many species, but the fundamental structural features of mandibular gear-like structures are likely conserved in adults, because they are the most suitable form for bark carving.

Functional gears are found rarely in animals, and in the single (Hemiptera: Issidae) previous report, nymphs of the planthopper *Issuscoleoptratus* had tiny rows of cuticular gear teeth (15–30 μm high) around curved medial surfaces of their two hindleg trochantera (Burrows and Sutton 2013). These gears of right and left trochantera are engaged during preparation for jumping and ensure that both hindlegs move at identical angular velocities to propel the body without yaw rotation. However, these gears occasionally fail to engage at the start of the propulsive phase of jumping due to separation before the jump. *Issus* gears also have only one direction of powered rotation and are lost during the final molt into adulthood. In contrast, the gear-like structures of horned beetles are comparatively large and heavily sclerotized, remaining completely engaged in two directions (open and close) throughout adulthood.

Multiple insect species are considered pests of living and dead or dying trees, and most bark beetles, such as *Pseudohylesinusnebulosusu* and *Dendroctonusponderosae* (Curculionidae), excavate egg galleries in fresh phloem. The locust borer *Megacyllenerobiniae* (Coleoptera: Cerambycidae) is a phloem wood insect that attacks living trees (Knight and Heikkenen 1980), and both adults and larvae bite the wood with their mandibles. In contrast, adult horned beetles carve living trees or wood with their mandibles. In the present Suppl. materials 2, 3, 7, the bark is hard and the xylem is soft like rotten wood that has been used for cultivation of mushrooms, and the adult *T.dichotomus* and *D.hercules* carved xylem more easily than bark. In agreement, photographs of adult horned beetles show that their heads are buried in more fibrous stem plants such as sugar cane (Unno 2006). These photographs indicate that adult horned beetles loosen the stem using the same action as that for bark-carving. Therefore, we propose the general use of the terms “tree-carving” or “plant-carving” to denote these carving behaviors of adult horned beetles.

## Supplementary materials

Supplementary material 1 Horned beetles Data type: Movie Explanation note: *Dynasteshercules* adult male and female and *Trypoxylusdichotomus* male. This is an open access article distributed under the terms of the Creative Commons Attribution License (CC BY 4.0), which permits unrestricted use, distribution, and reproduction in any medium, provided the original author and source are credited.Wataru Ichiishi, Shinpei Shimada, Takashi Motobayashi, Hiroaki Abe

Supplementary material 2 Bark-carving behaviors of *D.hercules*, adult males mainly from lateral side Data type: Movie This is an open access article distributed under the terms of the Creative Commons Attribution License (CC BY 4.0), which permits unrestricted use, distribution, and reproduction in any medium, provided the original author and source are credited.Wataru Ichiishi, Shinpei Shimada, Takashi Motobayashi, Hiroaki Abe

Supplementary material 3 Bark-carving behaviors of *D.hercules*, adult males mainly from anterior Data type: Movie This is an open access article distributed under the terms of the Creative Commons Attribution License (CC BY 4.0), which permits unrestricted use, distribution, and reproduction in any medium, provided the original author and source are credited.Wataru Ichiishi, Shinpei Shimada, Takashi Motobayashi, Hiroaki Abe

Supplementary material 4 Measurements of *T.dichotomus* mandible break resistance strengths under load Data type: Measurement This is an open access article distributed under the terms of the Creative Commons Attribution License (CC BY 4.0), which permits unrestricted use, distribution, and reproduction in any medium, provided the original author and source are credited.Wataru Ichiishi, Shinpei Shimada, Takashi Motobayashi, Hiroaki Abe

Supplementary material 5 Diagram of the direction (from the top to the bottom) of the load on mandible. View from the anterior Data type: Figure This is an open access article distributed under the terms of the Creative Commons Attribution License (CC BY 4.0), which permits unrestricted use, distribution, and reproduction in any medium, provided the original author and source are credited.Wataru Ichiishi, Shinpei Shimada, Takashi Motobayashi, Hiroaki Abe

Supplementary material 6 Determination of the presence or absence of gear-like structures on mandibles and types of tips in horned beetles and other insects Data type: Table Explanation note: a) +, presence; -, absence of gear-like structures. b) There was an individual variation. c) The chip of mandible was flat. d) The chip of mandible turned inward. This is an open access article distributed under the terms of the Creative Commons Attribution License (CC BY 4.0), which permits unrestricted use, distribution, and reproduction in any medium, provided the original author and source are credited.Wataru Ichiishi, Shinpei Shimada, Takashi Motobayashi, Hiroaki Abe

Supplementary material 7 Bark-carving behaviors of *D.hercules* adult females and a *Trypoxylusdichotomus* adult male Data type: Movie Explanation note: Horned beetles did not use the projection of clypeus but did use mandibles for bark carving. This is an open access article distributed under the terms of the Creative Commons Attribution License (CC BY 4.0), which permits unrestricted use, distribution, and reproduction in any medium, provided the original author and source are credited.Wataru Ichiishi, Shinpei Shimada, Takashi Motobayashi, Hiroaki Abe

Supplementary material 8 Synchronous movements of adult *D.hercules* mandibles Data type: Movie Explanation note: Movements of an intact head, a head after stripping the labium and maxillae, and a head after exposure of the gear structure; the gear-like structure was filed gradually to demonstrate the engagement point. Scenes were filmed from the ventral side. This is an open access article distributed under the terms of the Creative Commons Attribution License (CC BY 4.0), which permits unrestricted use, distribution, and reproduction in any medium, provided the original author and source are credited.Wataru Ichiishi, Shinpei Shimada, Takashi Motobayashi, Hiroaki Abe

Supplementary material 9 Comparison mandible muscles between *Trypoxylusdichotomus* horned beetles and the typical biting type insect *Locustamigratoria* Data type: Figure Explanation note: **A** anterior view of *L.migratoria***B** anterior view of male *T.dichotomus***C***L.migratoria***D***T.dichotomus*. To expose the mandible and its muscles the wall of the left half of the face was removed. Isolated mandibles with adductor and abductor muscles of *L.migratoria* (**E**) and *T.dichotomus* (**F**) Abbreviations: ad=adductor muscle; ab=abductor muscle. Scale bar 1 mm. This is an open access article distributed under the terms of the Creative Commons Attribution License (CC BY 4.0), which permits unrestricted use, distribution, and reproduction in any medium, provided the original author and source are credited.Wataru Ichiishi, Shinpei Shimada, Takashi Motobayashi, Hiroaki Abe

Supplementary material 10 The mouthparts of *Trypoxylusdichotomus* larva Data type: Figure Explanation note: **A** ventral view; **B** To expose the posterior regions of mandibles, the labium and maxillae were removed. When the mandibles were open, the hind regions of mandibles did not engage and no gear-like structure was observed.Abbreviations: Ant = antenna; Mx = maxilla; Lb = labium; Md = mandible; mol = molar lobe. Scale bar 2 mm. This is an open access article distributed under the terms of the Creative Commons Attribution License (CC BY 4.0), which permits unrestricted use, distribution, and reproduction in any medium, provided the original author and source are credited.Wataru Ichiishi, Shinpei Shimada, Takashi Motobayashi, Hiroaki Abe

Supplementary material 11 Movements of larval *Dynasteshercules* mandibles Data type: Movie Explanation note: When the larva bites a toothpick, both mandibles move synchronously, whereas manual closing of the left larval mandible was not accompanied by synchronous closing of the right mandible. This is an open access article distributed under the terms of the Creative Commons Attribution License (CC BY 4.0), which permits unrestricted use, distribution, and reproduction in any medium, provided the original author and source are credited.Wataru Ichiishi, Shinpei Shimada, Takashi Motobayashi, Hiroaki Abe

Supplementary material 12 Movements of adult *D.hercules* mandibles following breakage of gear-like structural regions Data type: Movie Explanation note: The mandibular gear region was broken using a file and mechanical synchronous movements were abolished. This is an open access article distributed under the terms of the Creative Commons Attribution License (CC BY 4.0), which permits unrestricted use, distribution, and reproduction in any medium, provided the original author and source are credited.Wataru Ichiishi, Shinpei Shimada, Takashi Motobayashi, Hiroaki Abe

Supplementary material 13 Synchronous movements of adult *D.hercules* mandibles Data type: Movie Explanation note: To expose the mandibles, the male head horn was removed and the cranium was filed as much as possible. Footage was filmed from the dorsal side. This is an open access article distributed under the terms of the Creative Commons Attribution License (CC BY 4.0), which permits unrestricted use, distribution, and reproduction in any medium, provided the original author and source are credited.Wataru Ichiishi, Shinpei Shimada, Takashi Motobayashi, Hiroaki Abe
