# Supplementary figures and images for: Completely engaged three-dimensional mandibular gear-like structures in the adult horned beetles: reconsideration of bark-carving behaviors (Coleoptera, Scarabaeidae, Dynastinae)
Source: Zookeys. 2019 Jan 7;(813):89–110. doi: 10.3897/zookeys.813.29236 (PMC6331513; doi:10.3897/zookeys.813.29236)

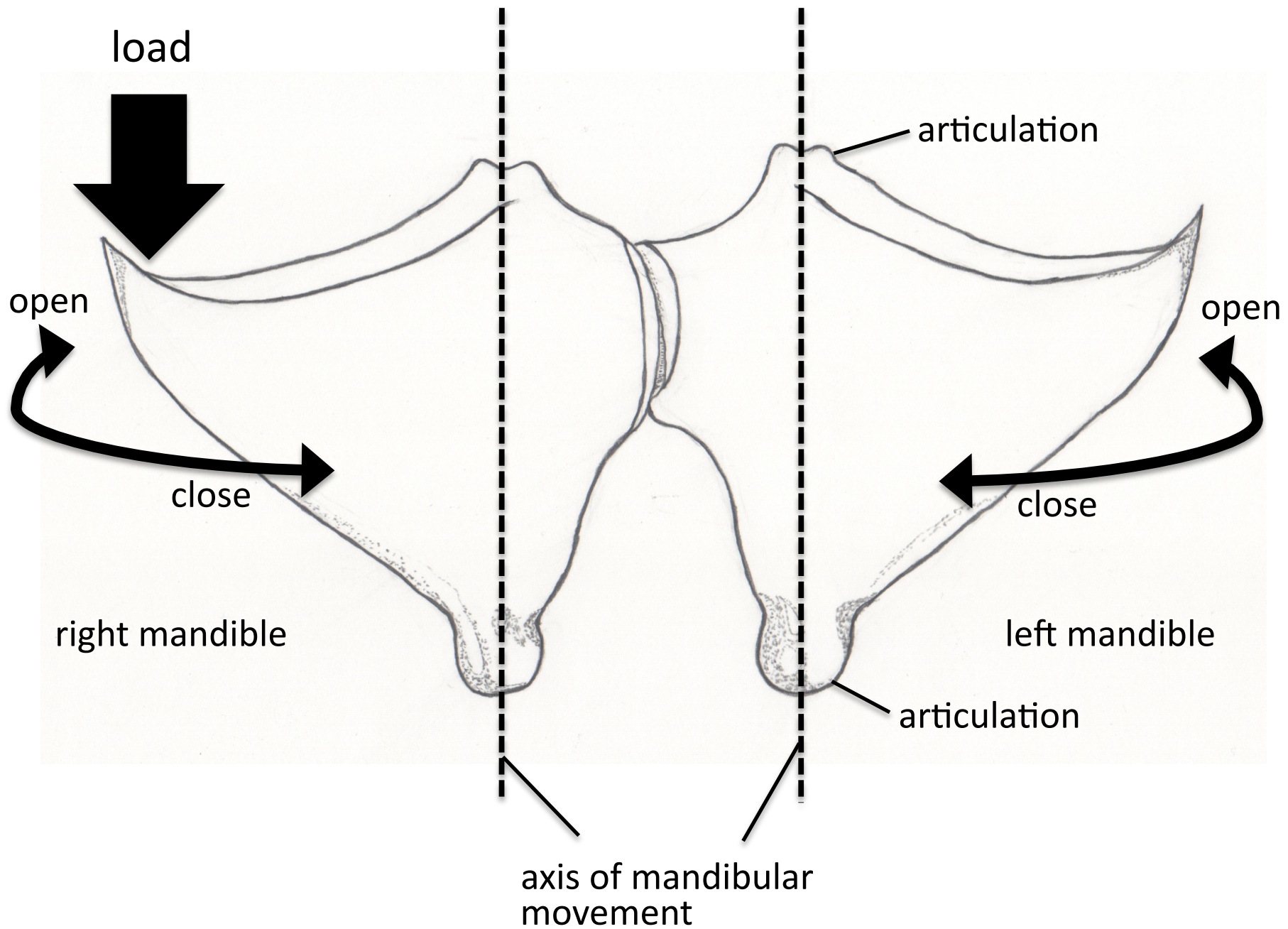

Supplement: Supplementary material 5 [file zookeys-813-089-s005.pdf]

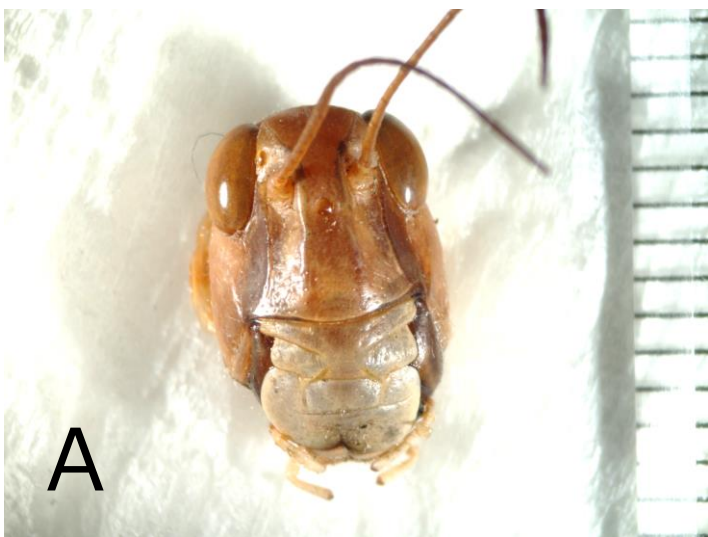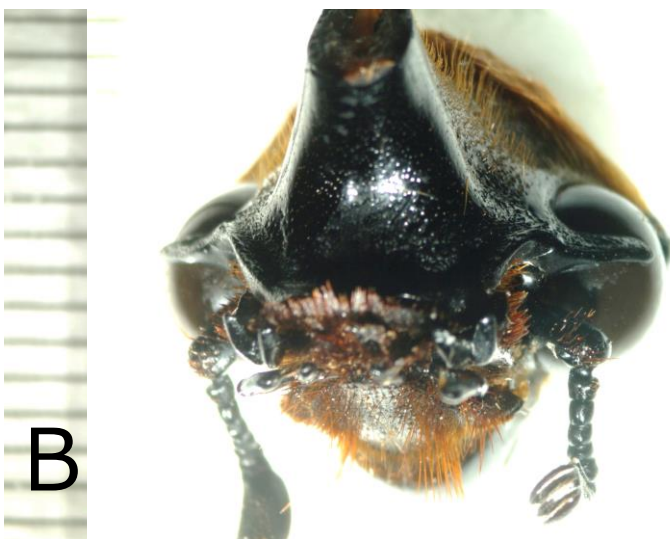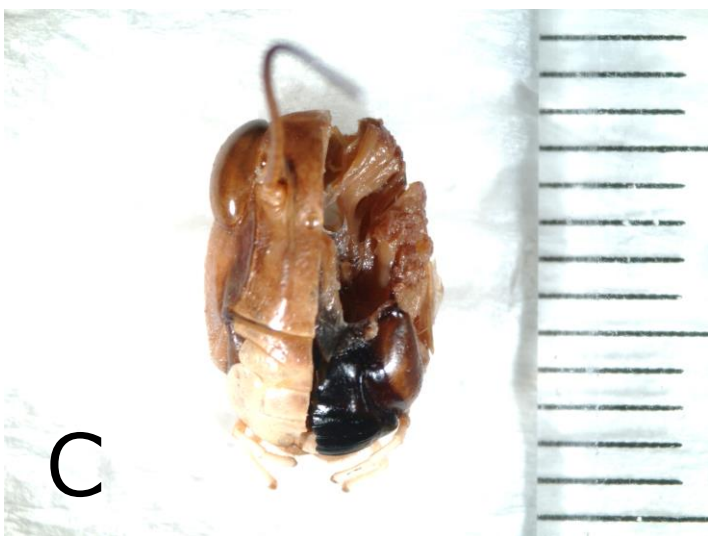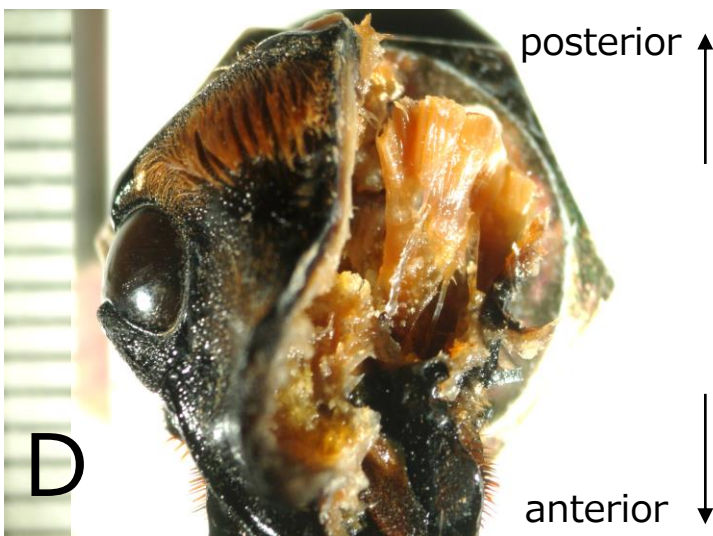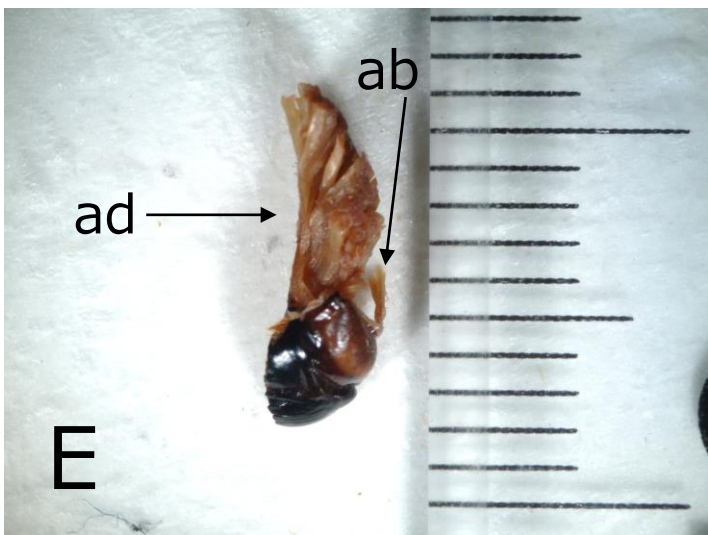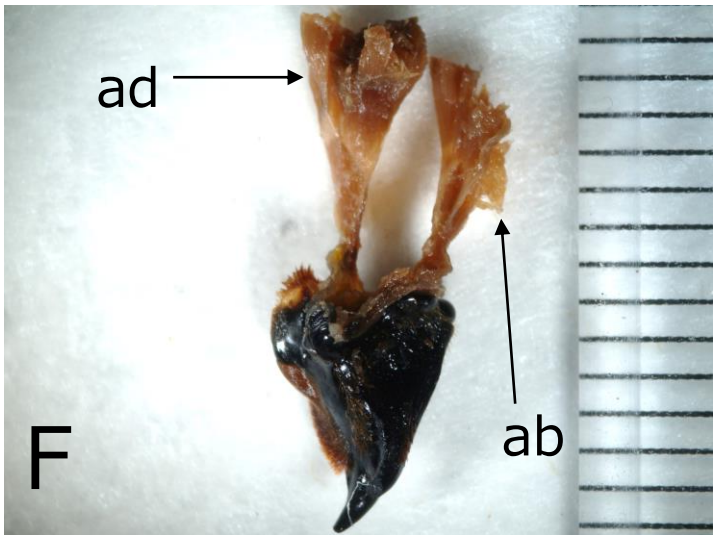

Supplement: Supplementary material 9 [file zookeys-813-089-s009.pdf]

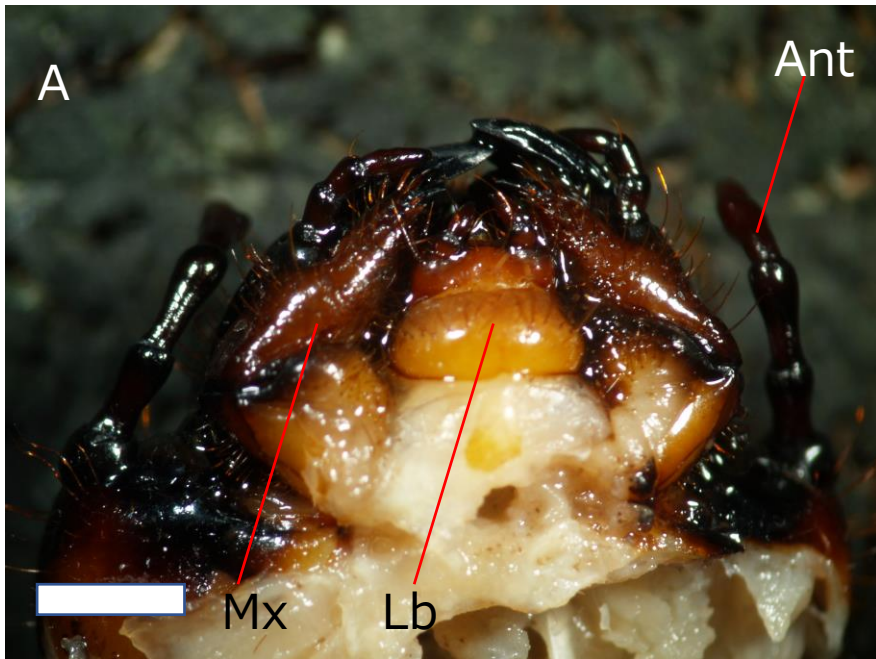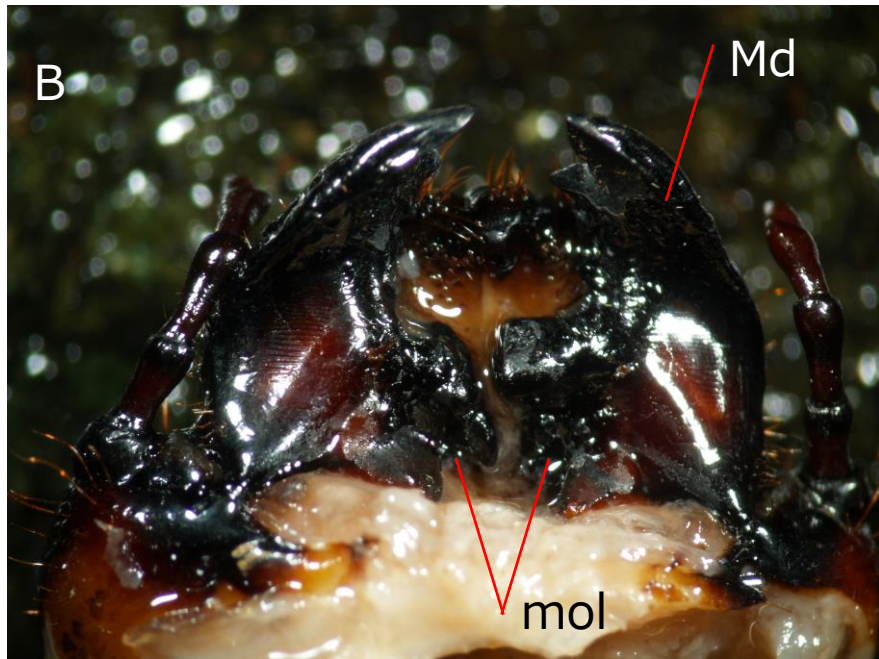

Supplement: Supplementary material 10 [file zookeys-813-089-s010.pdf]
